# Living single-cell metabolomics *via* mass spectrometry: state of the art and perspective

**DOI:** 10.1039/d6sc00482b

**Published:** 2026-04-22

**Authors:** Xianzhe Shi, Jiajun Peng, Chunxiu Hu, Muyingnan You, Xin Lu, Xinyu Liu, Guowang Xu

**Affiliations:** a Metabolomics Subcenter of the National Genomics Data Center, Dalian Institute of Chemical Physics, Chinese Academy of Sciences Dalian 116023 China xugw@dicp.ac.cn liuxy2012@dicp.ac.cn luxin001@dicp.ac.cn; b Liaoning Province Key Laboratory of Metabolomics Dalian 116023 China; c University of Chinese Academy of Sciences Beijing 100049 China

## Abstract

Cellular heterogeneity is an inherent feature of biological systems, and living single-cell metabolomics (SCM) has emerged as a powerful approach to probe this diversity—a dimension often lost in conventional bulk analyses. Currently, mass spectrometry (MS)-based living SCM techniques are driving a revolution toward higher throughput, sensitivity, and coverage, enabling the identification of rare cell subpopulations and expanding applications across various biological fields. Nevertheless, several bottlenecks remain, including limited metabolome coverage, insufficient throughput, batch effects, instrumental constraints, and challenges in processing large-scale datasets. Future efforts should focus on all stages of SCM, prioritizing the development of microfluidics-integrated living-cell analysis platforms, enhanced ionization sources, *in situ* chemical derivatizations, AI-powered data processing pipelines, and integrated multi-omics analyses at the single-cell level. Despite existing hurdles, continuous progress in technology, data science, and interdisciplinary collaboration is expected to bring transformative breakthroughs in MS-based living SCM, ultimately advancing our understanding of dynamic biological processes and accelerating biomedical discovery.

## Abstract 2

This perspective highlights advanced MS-based single-cell metabolomics, including analytical technologies, data processing, specialized devices, spatial metabolomics, and multi-omics. Future directions for living SCM are also discussed.

## Graphical abstract

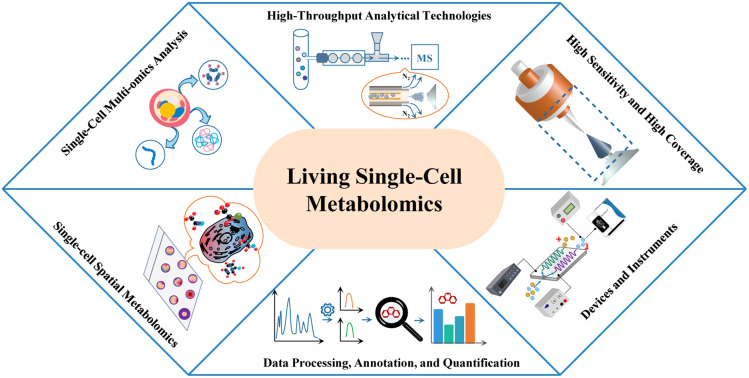

## 1. Introduction

Cellular heterogeneity is an inherent and fundamental characteristic in all biological systems, which can be induced by various genetic, epigenetic, stochastic biological processes and environmental factors.^1^ Moreover, cellular heterogeneity endows individual cells with distinct functional phenotypes, responses to stimuli, and fates, which plays a crucial role in various biological processes such as development, tissue homeostasis, immune response, and disease progression.^2,3^ Traditional bulk cell analysis which averages molecular signals across large cell population, frequently masks intercellular heterogeneity. In contrast, single-cell metabolomics (SCM) has emerged as a powerful tool to dissect cellular heterogeneity at the metabolic level.^4^

The overarching goal of SCM is to achieve comprehensive, unbiased, and quantitative characterization of metabolites within individual cells, thereby capturing the functional diversity of cell populations and providing mechanistic insights into biological processes. Its application scope is rapidly expanding, encompassing cancer biology, immunology, neuroscience, developmental biology, and precision medicine, where metabolic heterogeneity is increasingly recognized as a key driver of cellular behavior and therapeutic response. Notably, metabolites are highly dynamic and have rapid turnover rates, and ex vivo cell processing (*e.g.*, cell sorting, fixation, or lysis) often causes inevitable metabolic perturbation, leading to the distortion of authentic metabolic profiles that reflect *in vivo* physiological states. Only through living SCM analysis conducted within the native physiological microenvironment can researchers obtain real-time and accurate metabolic information.

In this context, “living SCM” is defined as the analysis of individual cells that are maintained in a viable state throughout the sampling and measurement process, without prior fixation permeabilization, or deliberate metabolic quenching. Such analyses aim to preserve the native or near-native physiological microenvironment of the cell, thereby enabling the acquisition of metabolic information that faithfully reflects dynamic *in vivo* states. This definition inherently excludes workflows that require cell fixation, drying, or offline chemical derivatization steps that perturb cellular activity, unless such steps are performed after controlled quenching that preserves the metabolic state at a defined time point. In the vast majority of cases, the studies presented in this perspective align with this definition, with only a few exceptions. These exceptions are included due to their unique research value.

Mass spectrometry (MS) is a versatile and powerful analytical technique that has become indispensable in SCM research.^5–7^ However, the extremely limited volume of individual cells poses enormous challenges for single-cell analysis. Since metabolites cannot be amplified and their physico-chemical properties in organisms can be widely diverse, MS-based technologies must possess ultra-high sensitivity and a broad detectable range to achieve comprehensive single-cell metabolome profiling. Additionally, high-throughput is imperative for single-cell analysis because a large number of single cells must be analyzed to obtain statistically significant results.

Owing to these technical hurdles, SCM remains in its nascent stage. Nevertheless, with continuous innovations in MS techniques, various MS-based platforms for single-cell metabolite profiling have emerged, offering powerful technical support for the development of this field and promoting its great potential in almost all biological disciplines.^8^ Therefore, this perspective focuses on cutting-edge MS-based strategies for single-cell metabolite analysis, encompassing high-throughput analytical technologies, high-sensitivity and high-coverage analytical platforms, data processing methodologies, and specialized devices/instruments tailored for SCM as well as single-cell multi-omics analysis. Finally, the future development direction and prospects of MS-based living SCM technologies are discussed.

## 2. State of the art of MS-based living SCM

The development of MS-based living SCM has undergone a progressive evolution driven by the pursuit of capturing metabolic heterogeneity and dynamic processes at the cellular level. In the early stage, MS-based SCM relied on capillary microprobes for manual aspiration of cytoplasm from living single cells under microscopic observation, and then the microprobes were directly served as nanoelectrospray ionization (nanoESI) emitters to introduce samples into MS for detection. A representative technology named living single-cell video MS (LSC-MS) enabled *in situ* analysis of living cells,^9^ and adjustable capillary tip sizes broadened its applicability to various cell types including animal and plant cells.^10^ To extend MS signal acquisition duration, pulsed direct current electrospray ionization MS (pulsed-dc-ESI-MS)^11^ and induced nanoESI (InESI)^12^ were developed, achieving prolonged signal detection from picoliter-volume single-cell samples. However, these early approaches suffered from low throughput due to laborious manual operations. Subsequently, flow cytometry-MS (FC-MS) emerged as a mainstream technology by integrating single-cell isolation, metabolite extraction, and ionization, significantly improving analytical throughput.

Notably, recent advancements in SCM have increasingly shifted toward living single-cell analysis, marking a pivotal transition from static profiling of fixed cells to real-time dynamic monitoring of viable cells under physiological conditions. This focus on living cells aims to minimize metabolic perturbations caused by ex vivo manipulation, thereby retaining the authentic metabolic state of cells. A general workflow of SCM typically includes five key steps: single-cell isolation, metabolite extraction, ionization, MS detection, and data processing including metabolite annotation. Methodological innovations in living-cell SCM have centered on optimizing sampling strategies and ionization efficiency to improve analytical sensitivity and throughput, thereby expanding metabolites' coverage at the single-cell level. Integration with microfluidic systems and hybrid ionization techniques has further advanced living single-cell analysis from low-throughput, low-coverage assays to high-throughput, high-precision detection, enhancing the efficiency and accuracy of metabolite characterization. In particular, MS imaging (MSI) techniques, such as matrix-assisted laser desorption ionization MS imaging (MALDI-MSI) and secondary ion MS (SIMS) imaging, enable the simultaneous acquisition of metabolic information and spatial coordinates, allowing *in situ* mapping of metabolite distributions within tissues and cellular microenvironments at single-cell or even subcellular resolution. Such spatial metabolomics approaches have become indispensable for investigating metabolic heterogeneity in its native tissue context, providing critical insights into cell–cell interactions, metabolic compartmentalization, and tumor microenvironment crosstalk.

The following sections will focus on the latest advancements in living SCM analysis, elaborating on technological breakthroughs, methodological innovations, and their applications in biological fields.

### 2.1 High-throughput analytical technologies

**Fig. 1:**
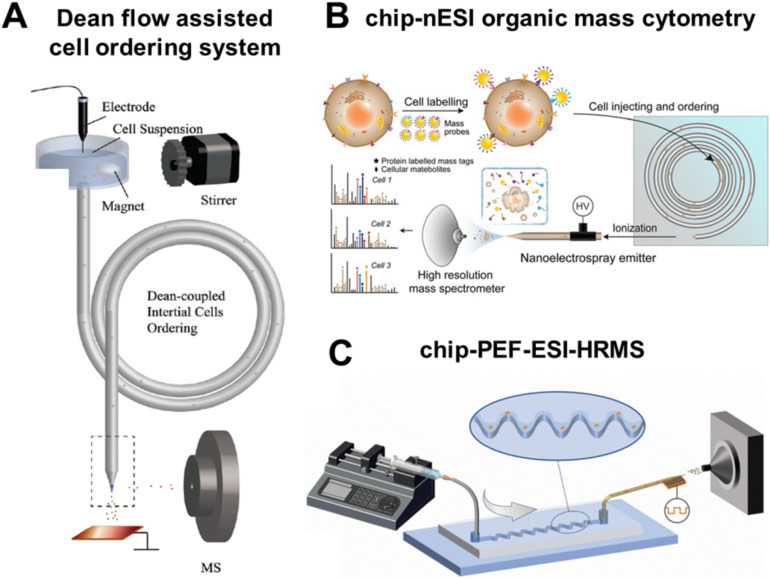
High-throughput single-cell analysis based on inertial microfluidics. (A) Schematic of Dean flow assisted cell ordering system for single-cell analysis. Reproduced from ref. 14 with permission from Royal Society of Chemistry, copyright 2018. (B) Schematic of the multi-dimensional chip-nanoESI organic mass cytometry. Reproduced from ref. 15 with permission from John Wiley and Sons Ltd, copyright 2021. (C) Schematic of asymmetric serpentine channel microfluidic chip coupled to pulsed electric field-induced ESI-HRMS. Reproduced from ref. 16 with permission from Elsevier, copyright 2022.

To achieve high-throughput SCM analysis, the first step is to realize high-throughput single-cell isolation from cell suspension. The inertial effect of Dean flow in microfluidic systems provides a simple and efficient design concept for obtaining single-cell streamlines. Wei *et al.* developed a single-cell focusing system with a 3D helical tube array as the core component, which drives cells to the equilibrium position through inertial lift and Dean resistance to form a single-cell flow.^13^ By using similar techniques, Lin's team induced Dean flow through helical capillaries to achieve ordered arrangement of single cells, reducing the uneven distribution and aggregation of cells in the cell suspension (Fig. 1A).^14^ In addition to capillaries, secondary flow can also be generated using microfluidic chips.

Xu *et al.* designed a microfluidic chip with five-turn helical channels and connected it to the nanoESI source, establishing a chip-nanoESI MS cytometry platform (Fig. 1B).^15^ It enables simultaneous monitoring 6 cell surface proteins and 84 metabolites at a throughput of approximately 40 cells per min. Our group designed a microfluidic chip with an asymmetric serpentine channel to disperse and focus cell suspension into a stable single-cell flow, which further increased the analytical throughput to 80 cells per min (Fig. 1C).^16^ Subsequently, this SCM approach was employed to the polarization research of macrophages. Glutamine was identified as a key metabolite that distinguishes the phenotypes of M1 and M2 macrophages.^17^

**Fig. 2:**
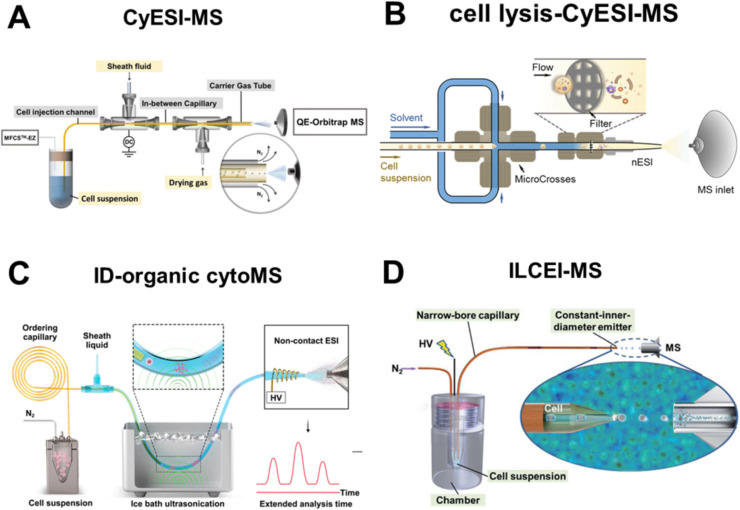
Flow cytometry-based single-cell high-throughput MS. (A) Schematic of CyESI-MS. Reproduced from ref. 18 with permission from American Chemical Society, copyright 2019. (B) Schematic of online cell lysis by zinc oxide nanothorn-decorated filters. Reproduced from ref. 24 with permission from American Chemical Society, copyright 2021. (C) Schematic of in-depth organic mass cytometry (ID-organic cytoMS). Reproduced from ref. 26 with permission from Springer Nature, copyright 2024. (D) Schematic of the ILCEI-MS system. Reproduced from ref. 27 with permission from Royal Society of Chemistry, copyright 2022.

In addition to the rapid and continuous single-cell injection achieved through inertial focusing, researchers have developed a single-cell MS method based on modified flow cytometry. These two approaches differ fundamentally in their separation mechanisms: Dean flow-based inertial focusing uses passive hydrodynamic forces within curved microchannels to align cells into a single streamline, relying on the balance of inertial lift and Dean drag forces, whereas modified flow cytometry relies on active hydrodynamic focusing in straight microchannels, enabling single-cell separation through the shearing of high-speed sheath fluid. Yao *et al.* coupled flow cytometry with ESI-MS (CyESI-MS) using three coaxial capillaries of different sizes for cell introduction, intracellular content extraction and solvent evaporation, respectively (Fig. 2A).^18^ CyESI-MS has been successfully applied to study chemoresistance heterogeneity in triple-negative breast cancer^19^ and lymphocyte subtypes,^20^ as well as dynamic shifts in metabolites^21^ and oxidized lipids^22^ in HepG2 cells following co-culture with natural killer (NK) cells, thereby demonstrating the potential of this technology in clinical research. Notably, single-cell lipid profiling revealed that co-culture with NK cells rapidly remodeled lipid oxidation pathways, as evidenced by the decreased levels of polyunsaturated lipids such as PC(38:6) and the corresponding increase in their oxidized forms, *e.g.*, PC(36:6-2OH). This provides direct evidence that immune cell engagement drives distinct metabolic reprogramming in target cancer cells, a finding that would be entirely obscured by bulk measurements due to the asynchronous nature of immune attack. Recently, a plug-and-play sample introduction device was developed for mass cytometry analysis. Integrated with a gas-driven flow focusing hole, this device generates a liquid jet to eliminate the need for a conventional nebulizer. Single-cell monodispersion is accomplished *via* liquid jet breakup, which effectively prevents clogging the thin central capillary.^23^

Promoting effective cell lysis and complete release of intracellular contents during the flow process is a key prerequisite for expanding the coverage of metabolites. However, in solvent-based extraction protocols, the interaction duration between cells and sheath fluid is merely a few seconds, which imposes inherent limitations on the efficacy of cell lysis and sample extraction. High-voltage electric fields, mechanical lysis and ultrasonic treatment are all effective means of cell lysis. Mechanical lysis based on sharp nanostructures can effectively rupture cell membranes and facilitate the release of intracellular contents. Xu *et al.* established a flow cytometry-MS system by integrating a filter modified with zinc oxide nanokines into nESI-MS for efficient online lysis of both cellular and nuclear membranes (Fig. 2B), which increased the signal response of certain intracellular metabolites by 2–11 fold.^24^ Nevertheless, the nanostructures employed for cell rupture are prone to contamination, necessitating washing after the analysis of approximately 100 cells. To address this limitation, the group further explored unmodified nanostructured small-bore filters, which increased the analytical throughput from 4 to 20 cells per min and enabled the batch analysis of 6000 cells in a single run.^25^ In addition, ultrasonic technology has been employed to facilitate the mixing of cell suspensions with sheath fluid for online cell lysis. Qin *et al.* developed an in-depth organic mass cytometry platform (Fig. 2C) to achieve ordered dispersion of single cells in capillaries through Dean flow. Upon exiting the capillary, the cell suspension is mixed with sheath fluid *via* ice-bath ultrasonication, and the resulting single-cell lysates are subsequently analyzed by non-contact ESI-MS. The online single-cell lysis and metabolite diffusion caused by ultrasonication extended the MS detection window of each cell to 25 seconds, enabling the acquisition of rich MS/MS spectra. This feature facilitates accurate metabolite identification and precise isomer differentiation, with 348 and 224 metabolites successfully annotated in positive and negative ion modes, respectively.^26^

It is widely recognized that the use of sheath fluid and sheath gas during electrospray ionization can lead to significant dilution of metabolites in a single-cell. Furthermore, sample ionization prior to entering the MS inlet can cause diffusion, dilution and ion loss in the atmosphere, thereby greatly reducing the sample collection efficiency. Directly introducing individual living cells into MS and achieving ionization *via* low-pressure high-temperature environments can enable dilution-free injection and highly sensitive MS detection, significantly enhancing sample utilization. Wang' team developed the technology of continuously introducing living single cells into MS, achieving a detection throughput of 51 cells per min and identifying 368 metabolites (Fig. 2D). The superior performance of this method lies in two key features: first, the utilization of fine-bore capillaries with an inner diameter marginally smaller than the cell diameter, which enables efficient single-cell separation and transport while eliminating sample dilution by sheath fluid or sheath gas; second, as the cells reach the capillary tip, they form single-cell droplets containing only a thin solvent layer through electrical emission and remain structurally intact throughout flight until ionization occurs in the ion transport tube of mass spectrometer, which maximizes the utilization rate of single-cell samples. High-throughput detection of over 4000 primary single cells was achieved from multiple mouse organs.^27,28^ Recently, Luo *et al.* developed an ion mobility resolution MS flow cytometry technology based on the living-cell electrolaunching ionization MS (ILCEI-MS).^29^ This approach integrates ion mobility (IM)-MS with high-throughput single-cell injection technology. By leveraging collision cross-section (CCS) values derived from IM, it enables rapid gas-phase separation of metabolite ions, achieving three-dimensional characterization of metabolites including *m*/*z*, CCS, and signal intensity, thereby overcoming the limitations of traditional MS flow cytometry that rely solely on *m*/*z* for single-dimensional detection. This work from Zhu's team has advanced the coverage of single-cell metabolomics to a new level,^29^ and the detection limit is as low as the attomol level, which is expected to solve the critical challenge of distinguishing functional isomers in the future. However, the current method is limited to detection under positive ion mode. Extending this technology to negative ion mode will enable coverage of a broader range of non-protonated metabolites, such as fatty acids and organic acids, thereby further enhancing the panoramic coverage of the single-cell metabolome.

In high-throughput mode, to balance the compatibility of MS and cell viability, it is often necessary to replace non-volatile PBS buffer with volatile isotonic solutions such as formamide or ammonium acetate. However, whether this solution exchange affects the metabolic status of cells remains a point of debate. Cheng *et al.* developed a label-free, high-throughput viability-informed single-cell MS technology by using glutathione (GSH) as the endogenous viability marker and PC 34:1 as the cell event marker, which can achieve the simultaneous detection of single-cell viability and metabolic profile.^30^ In addition, the high throughput of single-cell analysis and the shorter MS acquisition time for each cell imposes great demands on the scanning frequency of MS, which needs to be carefully considered.

### 2.2 High sensitivity and high coverage analytical technologies

Detection sensitivity and metabolite coverage are two crucial factors in SCM. Enhancing the ionization efficiency of MS ion source for metabolites with varying polarities represents a broadly applicable and highly effective strategy. The nanoESI source, characterized by its small nozzle aperture (approximately 1–10 µm in diameter) and low-flow spray characteristics, effectively reduces interference from salts and impurities in samples. This contributes to higher ionization efficiency and lower matrix effects, demonstrating its unique advantages in SCM. However, existing SCM technologies are limited by reliance on a single nanoESI ionization mechanism, which predominantly allows detection of highly abundant and readily ionizable polar metabolites. For the analysis of low-abundance, poorly ionizable, and non-polar metabolites in single cells, current MS approaches continue to face substantial challenges in achieving adequate detection sensitivity and comprehensive metabolite coverage. Atmospheric pressure chemical ionization (APCI) is a plasma-based ambient soft-ionization technique that shares mechanistic similarities with other open-access methods such as direct analysis in real time (DART), dielectric barrier discharge ionization (DBDI),^31^ and low temperature plasma (LTP) probe. These ionization techniques excel in ionizing weakly polar compounds, serving as an effective complement to ESI. Therefore, the development of hybrid ion sources integrating two or more ionization modes facilitates the coverage of a broad spectrum of analytes.

Several hybrid ion sources have been reported in the literature. For example, a dual non-contact nanoESI/nanoAPCI (nESI/nAPCI) source can operate in ESI, APCI, or electrophoretic separation modes, enabling direct analysis of biological fluids even in high-salt matrices.^32^ In a similar design, the dual ESI + APCI ionization source can be individually activated in ESI, APCI, or ESI + APCI modes by adjusting the direct current (DC) and alternating current (AC) voltages applied to the ion source.^33^ When coupled with thermal, laser or direct-desorption sampling, these sources enable efficient ionization of metabolites across a wide polarity range in both biological fluids and solid samples. However, nearly all existing hybrid sources have been designed for bulk sample, with very few adapted to the single-cell level. Miniaturizing and integrating multiple ionization mechanisms are therefore essential for achieving high-coverage SCM.

**Fig. 3:**
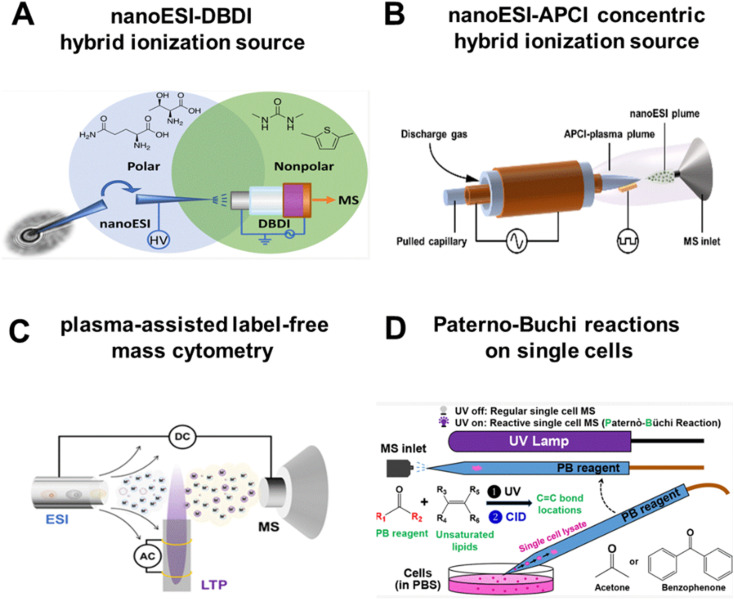
High sensitivity and high coverage analytical technologies. (A) Hybrid system combining nanoESI and DBDI. Reproduced from ref. 34 with permission from American Chemical Society, copyright 2022. (B) Concentric hybrid ionization source (nanoESI-APCI). Reproduced from ref. 35 with permission from Wiley-VCH Verlag, copyright 2024. (C) Schematic diagram of plasma-assisted label-free mass cytometry (PACyESI-MS). Reproduced from ref. 37 with permission from American Chemical Society, copyright 2024. (D) Paterno–Buchi reactions on single cells. Reproduced from ref. 38 with permission from American Chemical Society, copyright 2020.

Progress is already emerging on this frontier. The Zenobi group, for instance, developed a hybrid device by combining nanoESI with DBDI, where a capillary probe serves as both a sampling needle and a nanoESI emitter, while DBDI acts as a post-ionization source (Fig. 3A). With this setup, they detected 86 and 111 metabolites in an individual plant cell and in an animal cell, respectively.^34^ More recently, we designed a concentric nanoESI-APCI source that captures both polar and nonpolar metabolites in a single cell (Fig. 3B). In this design, a pulled glass capillary is coaxially inserted into a dielectric-barrier layer glass tube, so the two ionization mechanisms can be operated in perfect register. This hybrid source lowers the limit of detection (LOD) to 10 pg mL^−1^ (an order of magnitude gain), and obtained 254 annotated metabolites from a single cell, 82 more than nanoESI alone.^35^ Subsequently, we profiled 301 circulating tumor cells (CTCs) from patients and animal models. The platform identified 390 unique metabolites and revealed distinct metabolic fingerprints associated with different metastatic potentials (*e.g.*, brain *vs.* bone metastasis). These metabolic profiles enabled the construction of a classification model to stratify CTCs by metastatic risks.^36^ Clinically, this stratification demonstrated that CTCs from patients with brain metastasis exhibited abnormally elevated phenylalanine metabolism, whereas those from patients with bone metastasis showed activation of the arginine biosynthesis pathway, suggesting that metastatic organotropism is pre-encoded in the metabolic state of CTCs. Such capability to prospectively identify high-risk CTC subpopulations based on their metabolic signatures underscores the translational potential of high-coverage SCM in precision oncology. By combining the advantages of orthogonal hybrid ionization, a plasma-assisted label-free mass cytometry (PACyESI-MS) was proposed for high-throughput single microalgae analysis.^37^ PACyESI-MS achieves a throughput of up to 52 cells per minute, while enabling the simultaneous detection of dozens of critical primary and secondary metabolites within individual microalgal cells (Fig. 3C).

Beyond hybrid ion sources, coupling MS with chemical reactions represents another effective strategy to enhance metabolite detection sensitivity. Zhu *et al.* utilized a glass micropipette needle to perform Paterno–Buchi (PB) reactions on single cells (Fig. 3D). With the assistance of tandem MS (MS/MS) analysis, this approach enabled the determination of carbon–carbon double bond (C=C) positions in unsaturated lipids at the single-cell level.^38^ In another study, Li *et al.* developed a shotgun MS protocol for high-specificity single-cell lipidomics, including the determination of both C=C locations and sn-positions. Cell fixation and batch photochemical derivatization were employed to identify C=C locations and sn-positions of lipids, respectively. By quantifying lipid C=C or sn-position isomers, the platform successfully classified four subtypes of human breast cancer cells.^39^ Subsequently, the same group integrated single-cell fixation, PB derivatization, and CyESI-MS to develop a high-throughput single-cell structural lipidomics platform, structurally characterizing 145 lipid subclasses and quantifying 17 isomeric lipids differing in C=C bond locations.^40^

Similarly, to target low-abundance metabolites, Zhuang *et al.* designed a quaternary ammonium salt group-based charge tag that can be attached to cysteine *via* a biocompatible click reaction. When combined with induced nanoESI-MS, this strategy significantly enhanced the detection sensitivity of cysteine in single cells. Consequently, the low intracellular cysteine levels in single living HeLa cells were successfully quantified and dynamically monitored under pharmacological inhibition of cystine transport.^41^

Although technological innovations in ionization and chemical derivatization are rapidly advancing the field, several challenges still remain. The design of hybrid sources must carefully balance sensitivity, resolution, and compatibility with living cell sampling. In addition, translating bulk-scale derivatization chemistry to the single-cell level requires precise control of reaction conditions at a picoliter volume. Therefore, future efforts should focus not only on expanding the coverage of metabolites but also on improving quantitative robustness and throughput, ultimately achieving dynamic metabolic tracking of individual cells in their native microenvironment.

### 2.3 Devices and instruments for SCM analysis

**Fig. 4:**
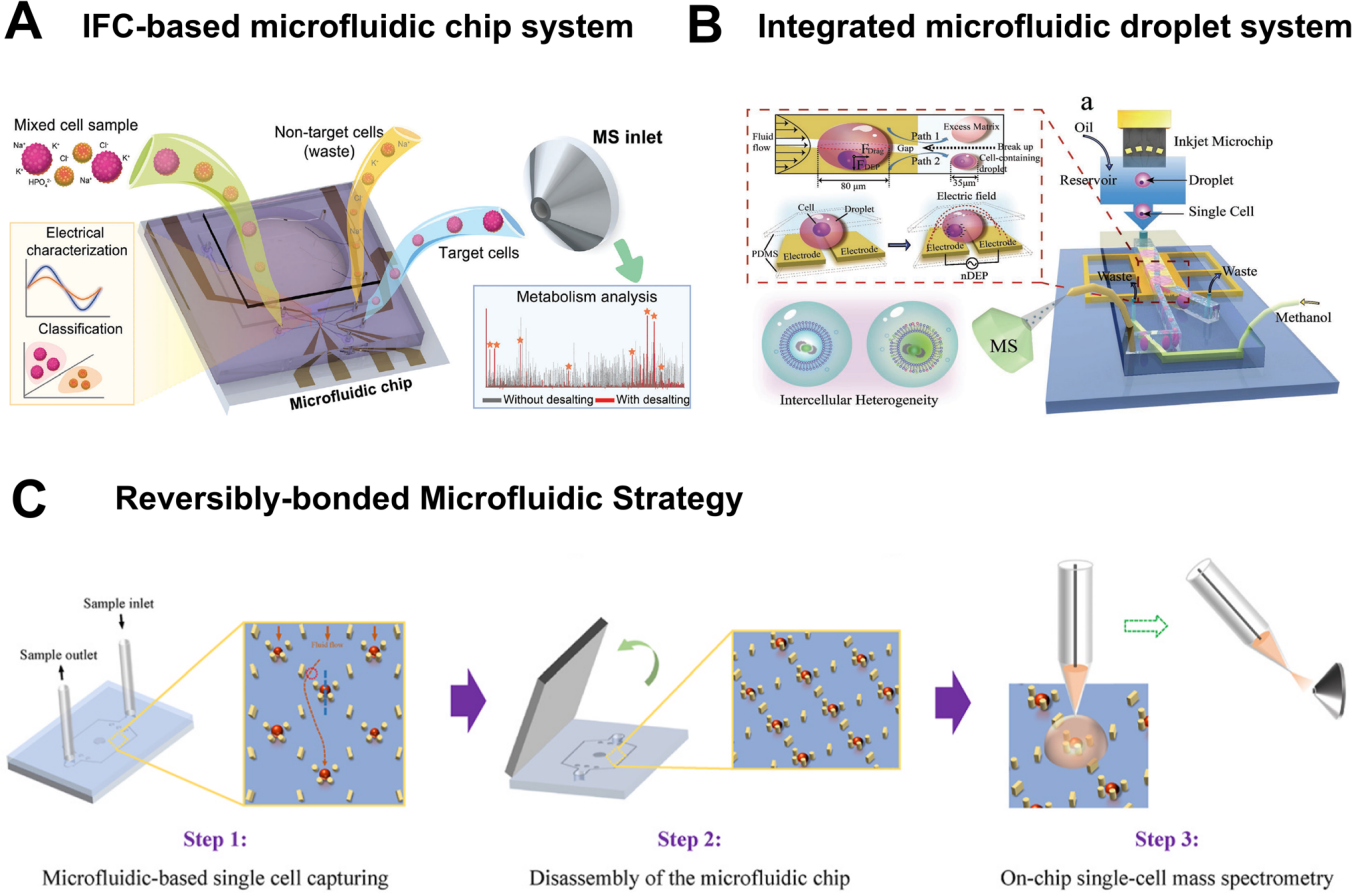
Microfluidic devices for SCM analysis. (A) IFC-based microfluidic chip for multimodal cell characterization. Reproduced from ref. 42 with permission from Wiley-VCH Verlag, copyright 2024. (B) Concentrating single cells in picoliter droplets for phospholipid profiling on a microfluidic system. Reproduced from ref. 44 with permission from Wiley-VCH Verlag, copyright 2020. (C) Streamlined on-chip single cell MS enabled by a reversibly-bonded microfluidic strategy. Reproduced from ref. 45 with permission from Elsevier, copyright 2025.

Due to the extremely small size of single cells and the particularity of the samples, specialized devices and instruments are often required for SCM analysis. Microfluidic technology, with its advantages of miniaturization and integration, has become the core technical path to solve the sample pretreatment problems in SCM, and has also promoted the development of various specialized integrated devices. Zhu *et al.* proposed an inertial focusing chromatography (IFC)-based microfluidic chip that achieves one-step single-cell sorting and desalination, thereby providing high-purity samples with minimal matrix interference for downstream MS analysis (Fig. 4A).^42^ IFC characterizes the label-free electrical properties of cells, and target cells are then transferred from high-salt buffer into MS-compatible ammonium formate solution *via* the PZT actuator, simultaneously accomplishing sorting and desalination. This chip can serve as an independent pretreatment module compatible with multiple MS techniques. However, cell sorting relies on differences in electrical properties, resulting in sufficient resolution for distinguishing cell subpopulations with similar electrical phenotypes.

Beyond applications in cell sorting and pretreatment, specialized sampling devices have been developed to enable precise, spatially resolved analysis of subcellular regions and to facilitate controlled handling of single cells with minimal perturbation. These tools are particularly valuable for probing metabolic heterogeneity within subcellular compartments and for integrating sampling with downstream MS analysis. For instance, Zhang *et al.* developed a microfluidic probe-based sampling system that overcomes the limitations of traditional methods by allowing precise extraction of insoluble components from subcellular region of living cells.^43^ Laminar flow was formed by using microfluidic probes, and the position and width of the sampling area (as small as 3.5 µm) were precisely controlled by regulating the flow rate of sampling solvent, enabling targeted extraction of insoluble components from organelles such as mitochondria, endoplasmic reticulum, and nucleus. Nevertheless, the sampling process can cause irreversible damage to the cells, and only the surface or near-surface components can be extracted. Zhang *et al.* developed an integrated microfluidic droplet system that achieves picoliter droplet encapsulation, concentration, and online MS analysis of single cells (Fig. 4B).^44^ However, limitations remain including inadequate droplet stability, poor encapsulation efficiency, and throughput constrained by MS response speed. You *et al.* developed a reversibly-bonded PDMS-PS double-layer microfluidic chip that enables seamless integration with the SCMS workflow (Fig. 4C).^45^ This chip remains bonded during cell isolation, and can be disassembled to expose the captured cells into ambient environment for on-chip SCMS analysis.

Traditional single-cell analysis is characterized by low operational efficiency and poor repeatability. The development of automated SCM analysis devices is a very promising direction. Chen *et al.* developed the first fully automatic single-cell MS analysis platform without human intervention.^46^ Its core design integrates robot micro-operation with sub-nanoliter droplet extraction technology. A microscopic imaging module provides visual feedback for precise positioning and enables operations such as generating sub-nanoliter droplets, extracting cellular extracts, and ionization. MS detection module employs a linear ion trap mass spectrometer and supports MS^1^ and MS^2^ analyses. To minimize dilution of intracellular metabolites, this system generates sub-nanoliter droplets with precise controlling over pipette tip radius, injection pressure and lifting speed. Meanwhile, the system incorporates a point-scanning algorithm to achieve 1 µm positioning accuracy of the pipette tip along the *X*–*Y* axes, and an automatic region-of-interest (ROI) algorithm for *Z*-axis focusing, which avoids tip positioning deviations associated with traditional full-image focusing. This platform improves single-cell analysis efficiency to 2 minutes per cell and eliminates inter-operator variability. However, its throughput is restricted by image recognition and visual servo stability, and the pipettes are prone to clogging without an automatic replacement device. Zhao *et al.* constructed an automated micro-picoliter extraction system that enables precise control of picoliter-scale solvents for efficient extraction of single-cell metabolites.^47^ This system utilizes PDMS microwell chips as cell capture carriers, integrating customized imaging, bus control and fluid drive modules. It is fully portable and compatible with various mass spectrometers. In terms of automation, the system adopts a Naive Bayes algorithm combined with GLCM features for single-cell identification, and the MOG2 moving object detection algorithm to accurately locate the pipette tip. Solvent volume is controlled based on the surface tension principle. This system improves metabolite detection sensitivity by 27.7% and achieves an analysis throughput of 20 cells per hour.

Most of the current instruments for SCM analysis remain at the laboratory-built stage, with only a few commercialized instruments such as SinCell-100, currently available. These instruments play a pivotal role in enhancing the throughput and robustness of single-cell analysis. With continued advancements in artificial intelligence and automation, an increasing number of single-cell analysis instruments are expected to be commercialized.

### 2.4 Data processing, annotation, and quantification in living SCM

**Fig. 5:**
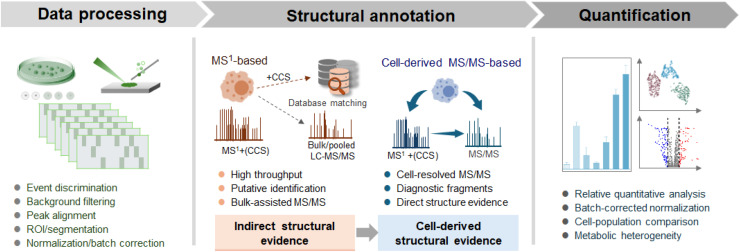
Data processing, annotation, and quantification in living SCM.

Data processing in living SCM is a necessary step because raw single-cell signals are sparse, heterogeneous, and often strongly affected by background fluctuations. Unlike bulk cell workflows, where population averaging masks stochastic variability, direct sampling of living single cells is often event-driven and time-resolved, making them highly susceptible to both technical and biological variability.^48^ Conventional pipelines are often insufficient for living single-cell data, and more adaptive processing strategies are needed to distinguish biologically meaningful variation from the sparsity and environmental noise of single-cell MS data (Fig. 5).^49^

A major challenge in sampling-based living single-cell analysis is the dynamic background, as the boundary between cellular signals and the extracellular environment exists within a fluid under continuous acquisition.^50^ Conventional background subtraction approaches may be inadequate, as they inadvertently eliminate biologically relevant signals, especially those arising from transient secretion or exchange processes. Recent workflows have increasingly adopted time-resolved filtering or scan-selection strategies to distinguish short-lived cellular signals from gradual baseline drift in the surrounding environment. In addition to signal extraction, feature alignment in high-resolution (HR) MS represents another trade-off. Binning-based alignment reduces computational complexity but may compromise mass accuracy, whereas alignment methods relying on high mass precision are more sensitive to stochastic peak shifts and isobaric interference under low signal-to-noise conditions. Systematic technical variation introduced by instrumental drift requires careful attention in living single-cell experiments. Normalization strategies should account for batch and run-order effects to reduce technical artifacts while preserving biologically meaningful variation across cell subpopulations.^51,52^

In living SCM, metabolite structural annotation is more challenging than in bulk or fixed-cell analyses. MS^1^-based annotation, often supported by bulk or pooled liquid chromatography-MS/MS (LC-MS/MS) data, remains the most practical strategy in current living SCM workflows owing to its reasonable throughput and feasibility under conditions of limited direct single-cell MS/MS acquisition.^53,54^ Within this framework, HR MS^1^ measurements are combined with database matching to generate putative annotations, providing a scalable solution for relatively abundant metabolites and broader chemical class assignment.^35,55,56^ Fundamentally, this remains an MS^1^-based annotation strategy, with MS/MS from pooled samples providing complementary rather than cell-resolved structural evidence.

Compared with indirect annotation supported by bulk measurements, single-cell MS/MS can provide more direct structural evidence, with tandem mass spectra strengthening metabolite assignments. Recent methodological advances have demonstrated the technical feasibility of direct single-cell MS/MS acquisition. When experimental workflows are specifically designed to accommodate the limited analysis time and ion yield of single-cell events, tandem mass spectra can provide substantially stronger support for metabolite annotation.^26,39^ Two representative strategies illustrate how direct single-cell MS/MS can be implemented under distinct analytical priorities.^26,39^ One single-cell lipidomics approach emphasizes structural specificity, particularly for chemically complex lipid species. By combining cell stabilization with chemically encoded fragmentation schemes such as PB reaction, lipid fine structures can be resolved, including C=C positions and acyl-chain regiochemistry.^39^ The resulting diagnostic fragment ions enable discrimination and relative quantification of lipid isomers that cannot be resolved by accurate mass or MS/MS alone. Using this method, a total of 145 lipids were structurally characterized at the subclass level, and the relative abundances of 17 C=C location isomers from 5 phosphatidylcholine (PC) precursors were determined in single cells. Fine structural characterization of lipids uncovers cell-to-cell heterogeneity masked by bulk analyses. For instance, resolving PC 18:1_18:1 C=C positional isomers enabled clear discrimination between breast cancer and normal cells, while correlation analysis of lipid isomers across PC species reflected distinct desaturase activities in cancer cells. These results underscore that lipid isomer-level resolution is essential for revealing metabolic heterogeneity and disease-associated remodeling. A complementary single-cell metabolomics strategy emphasizes MS/MS acquisition depth and coverage. By integrating continuous-flow single-cell MS platforms with efficient online cell lysis, this approach extends the effective analysis time per cell, substantially increasing the number and diversity of MS/MS spectra from a single-cell event.^26^ The system allows repeated high-resolution MS^1^ scans and data-dependent MS/MS acquisition over tens of seconds, enabling identification of diverse small molecule classes, including central carbon metabolites, organic acids, fatty acids, and lipids. Direct observation of diagnostic fragment ions at the single-cell level supports isomer discrimination and relative quantification with substantially reduced reliance on pooled or bulk reference spectra.

Orthogonal structural information can further strengthen annotation. IM-MS provides CCS values as an additional descriptor of molecular structure, which is particularly valuable for resolving lipid isomers.^57,58^ CCS values can serve as complementary constraints that reduce candidate structures when direct MS/MS evidence is limited. Recent ion mobility-resolved single-cell metabolomics workflows combine MS^1^ matching, CCS constraints, and targeted single-cell MS^2^ acquisition to strengthen metabolite annotation.^29^ This design extends the structural coverage beyond conventional MS^1^-based workflows by incorporating ion mobility and consensus MS^2^ spectra into the annotation process. Even so, these allocations are still established partly through candidate matching with the reference database, and the strongest structural support comes from spectra aggregated across cells rather than from independent MS^2^ evidence acquired from each individual cell.

Quantification in living SCM remains more difficult. Absolute quantification in sampling-based single-cell analysis remains challenging because it requires reliable estimation of cell volume, capture efficiency, and ionization efficiency. For these reasons, most current living SCM studies rely predominantly on relative rather than absolute quantification. Normalization and calibration are essential to mitigate run-order-dependent drift in living SCM.^59,60^ Normalized intensity measures, including total ion current scaling, reference-ion normalization, and feature-wise scaling, are routinely applied to improve comparability. Batch correction is also required for quantitative comparisons under multi-batch acquisition. In targeted or semi-targeted settings, relative quantification of predefined metabolite features further enables population-level comparisons and trend analysis.

### 2.5 Single-cell spatial metabolomics

The spatial distribution of metabolites within cells is a key factor in interpreting cellular heterogeneity. Single-cell spatial metabolomics is a cutting-edge omics technology that enables unbiased, global, and quantitative characterization of metabolites in biological tissues or cellular microenvironments under the dual constraints of single-cell resolution and *in situ* spatial coordinates. At present, this field is mainly based on MS imaging techniques, such as SIMS imaging, MALDI-MSI and so on. Zhang *et al.* employed MALDI-MSI to successfully distinguish the metabolic heterogeneity of a variety of tumor cells with a lateral resolution of 20 µm. They further revealed the differential expression and interaction patterns of metabolites after co-culture between tumor cells and fibroblasts.^61^ Delafiori *et al.* developed a high-throughput SCM method named HT SpaceM by combining cell preparation on custom glass slides with MALDI-MSI, which enables robust, large-scale SCM across over 140 000 cells from 132 samples.^62^ Rovira-Clavé *et al.* developed a high-definition multiplex ion beam imaging technology based on the cesium ion beam SIMS platform, improving the resolution to subcellular nanoscale levels with lateral and axial resolutions of approximately 30 nm and 5 nm, respectively.^63^ Pareek *et al.* successfully established a 3D SIMS single-cell imaging technology to reveal the metabolic channeling function of purine bodies.^64^

Despite these advances, traditional SIMS can disrupt the intrinsic distribution, localization, and chemical properties of biomolecules by operating in a vacuum environment at the expense of sample integrity. To address this, Lim *et al.* developed a graphene-based SIMS imaging technology that allows direct analysis of wet cells while maintaining cell viability.^65^ In this approach, prepared graphene is floated on the surface of a 75 mM ammonium acetate solution to flatten it, and cells are then brought into contact with the floating graphene monolayer, which automatically tiles and adheres tightly to the cell surface. After mild drying at room temperature for 10 minutes, the coated samples are rapidly transferred to an ultra-high vacuum for TOF-SIMS analysis. After graphene coating, cells exhibited no membrane rupture and showed no significant morphological or intracellular structural changes within 5 minutes of vacuum exposure and up to 60 minutes of total coating time. Using this *in situ* imaging method, they directly observed the heterogeneous distribution of cholesterol on untreated wet cell membranes. However, it is important to note that this graphene-based SIMS imaging technique has not yet been applied to metabolomics.

Apart from MS imaging technology, the data processing challenges often involve cell segmentation, region-of-interest (ROI) definition, and the separation of spatially mixed signals prior to cell-level feature extraction. Errors in segmentation or ROI definition directly affect the accurate assignment of ion signals to individual cells.^66^ Signal attribution is further complicated by spatial spillover, mixed pixels at cell boundaries, and interference from adjacent cells or the surrounding matrix, all of which can introduce ambiguity in source identification and compromise the specificity of single-cell measurements. In imaging-based workflows, quantitative variability can also arise from mixed pixels at cell boundaries, inaccuracies in segmentation or ROI definition, local differences in microregion sampling conditions, and the conversion of pixel-level signals into cell-level measurements. These factors collectively contribute to inconsistencies in signal quantification and may obscure true biological heterogeneity. Accordingly, normalization in imaging-based single-cell analysis may need to account for segmented cell area, local matrix effects, and cell-to-cell differences in morphology or sampled volume, ensuring that downstream analyses reflect biological rather than technical variations.

### 2.6 Single-cell multi-omics analysis

**Fig. 6:**
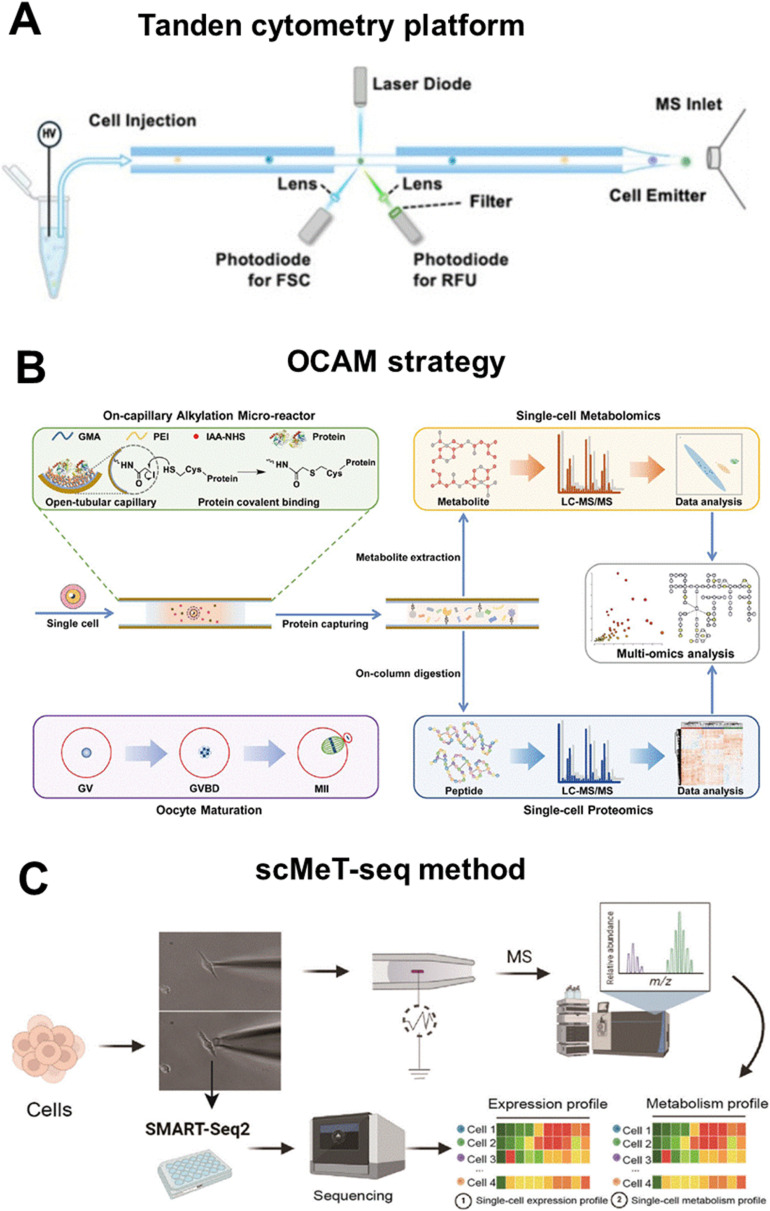
Overview of representative methods for single-cell multi-omics analysis with the metabolomics as the core: (A) schematic of tandem cytometry platform combining fluorescence flow cytometry with NanoESI-MS for one-step analysis of proteins and metabolites in single cells. Reproduced from ref. 67 with permission from American Chemical Society, copyright 2025. (B) Schematic of the OCAM strategy for proteo-metabolome profiling in the same single cells. Reproduced from ref. 70 with permission from Royal Society of Chemistry, copyright 2023. (C) Schematic of the procedure of scMeT-seq method. Reproduced from ref. 72 with permission from Wiley-VCH Verlag, copyright 2025.

Broadly speaking, current single-cell multi-omics approaches can be classified into three main categories based on their technical strategies. The first category targets cell-surface proteins and metabolome by converting protein abundance into either MS-detectable reporters or fluorescence signals *via* mass tags or labelled antibodies. Xu *et al.* developed a multi-dimensional organic mass cytometry system that integrates a microfluidic chip for cell dispersion with NanoESI-MS for ionization and detection.^15^ A series of mass probes (MPs) were constructed by self-assembling rhodamine-based mass tags (RMTs) and target-specific antibodies onto gold nanoparticles (GNPs). While antibodies enabled specific recognition of cell-surface proteins, RMTs dissociated during NanoESI, converting protein abundance into amplified, interference-free MS reporters for semi-quantification. This integrated platform simultaneously profiled six cell-surface antigens and ∼100 metabolites at a throughput of ∼40 cells per minute. Complementarily, Du *et al.* established a tandem cytometry platform combining fluorescence flow cytometry with NanoESI-MS for one-step analysis of proteins and metabolites in single cells (Fig. 6A).^67^ Target proteins were labelled with fluorescent antibodies, and a high-temporal-resolution laser-induced fluorescence (LIF) system recorded single-cell events *via* forward scattering (FSC) and relative fluorescence units (RFU) for protein quantification. The same cells were subsequently injected through a constant-inner-diameter emitter for NanoESI-MS, enabling untargeted detection of ∼300 metabolites.

Zhao *et al.* constructed an automated multi-omics sample pretreatment system centered on a 10 pL-level PDMS microwell chip, which captures single cells and enables rapid enzymatic hydrolysis of proteins. This system detects 1391 substances including 651 proteins, 524 phospholipids, and 216 metabolites from a single cell within 10 minutes. In the analysis of clinical tissue samples from liver cancer, lung cancer, and breast cancer, the classification accuracy achieved with multi-omics markers was 15% to 40% higher than that with single-omics markers.^68^ Notably, the integration of multi-omics data enabled better differentiation of cancer cell subtypes and improved clustering performance, demonstrating that combining metabolites, phospholipids, and proteins provides a more comprehensive phenotyping capacity than any single omics layer alone.

The second category shifts focus to intracellular proteins and metabolites, achieving simultaneous profiling through direct detection of endogenous molecules following physical or chemical separation *via* capillary electrophoresis, chemical immobilization, or chromatographic separation. Lombard-Banek *et al.* developed an *in vivo* subcellular HR MS platform that enabled simultaneous proteo-metabolomic analysis of spatiotemporally defined single cells within living *Xenopus laevis* embryos.^69^ Light microscopy localized target cells in cleavage-stage embryos, and precision-translated microcapillaries were used for rapid dual micro-aspiration of subcellular content, removing <5% of cell volume to preserve embryonic integrity. The aspirates were analyzed by a custom capillary electrophoresis-ESI (CE-ESI) system coupled with Orbitrap or time-of-flight HR MS, allowing label-free detection of ∼150 metabolites and 738 proteins without prior knowledge of cell composition. He *et al.* reported an on-capillary alkylation micro-reactor (OCAM) strategy for concurrent proteo-metabolomic profiling from the same single cell (Fig. 6B).^70^ Individual cells are lysed within the OCAM, where proteins are covalently immobilized on an iodoacetic-acid-functionalized capillary wall *via* sulfhydryl alkylation, minimizing protein loss and permitting the use of detergents such as SDS. Metabolites are rapidly eluted with a high-concentration organic solvent for untargeted LC-MS/MS analysis, while immobilized proteins undergo on-column digestion with trypsin/Lys-C for subsequent proteomic detection. This “one-pot” workflow eliminates sample transfer, enhances recovery, and avoids cross-interference between omics, enabling identification of 3457 protein groups and 171 metabolites in single mouse oocytes. Similarly, Wu *et al.* developed a one-shot single-cell proteome-and-metabolome analysis (scPMA) strategy for simultaneous profiling of proteins and metabolites within a single LC-MS/MS run.^71^ Target cells are captured *via* a capillary probe and pretreated in micro-insert tubes, after which the sample is injected into a nanoLC system with a C18 column for sequential separation of enzyme-digested peptides and relatively hydrophobic metabolites. A dual-zone MS detection mode optimized independently for peptides and metabolites enables comprehensive identification, yielding label-free detection of up to 816 proteins and 148 metabolites per single cell.

The third category integrates metabolomic and transcriptomic profiling within the same viable cell through dual-step capillary aspiration that sequentially extracts cytoplasmic sub-volumes for MS and the remaining cell for high-sensitivity RNA sequencing. Mao *et al.* developed a single-cell simultaneous metabolome and transcriptome profiling (scMeT-seq) method, allowing coordinated analysis of both omics layers from the same living cell (Fig. 6C).^72^ A nano-capillary with a 300 nm opening aspirates sub-picoliter volumes of cytoplasm for metabolomic analysis, preserving cell viability while providing sufficient analytes for ESI-MS detection of ∼100 metabolites across key pathways such as glycolysis and TCA cycle. Immediately thereafter, a 9 µm capillary containing lysis buffer extracts the intact cell for transcriptome sequencing *via* a modified SMART-seq2 protocol, yielding an average of 7424 genes per cell, comparable to standalone scRNA-seq.

Taken together, these technological advancements demonstrate the feasibility of integrating multi-omics layers at the single-cell level, with metabolomics as a core analytical component, and highlight the potential of MS as a unified platform for advancing single-cell systems biology. However, sample pretreatment protocols and MS detection methods vary substantially across omics disciplines, posing significant challenges for simultaneous single-cell multi-omics profiling. Furthermore, the effective integration of heterogeneous single-cell multi-omics datasets is another critical bottleneck in this rapidly developing field.

## 3. Future directions and perspectives

MS-based living SCM has emerged as a powerful approach in biological research, opening up new avenues for revealing cellular heterogeneity and metabolic diversity. By enabling direct metabolite profiling in individual cells, this technology provides deep insights into the metabolic signatures of distinct cell (sub) types and their adaptive responses to physiological or environmental stimuli. Current state-of-the-art platforms encompass a diverse range of high-throughput, high-sensitivity, and high-coverage analytical technologies. Label-free mass cytometry, for instance, allows the analysis of hundreds of cells per experiment and facilitates the identification of rare cell subpopulations. Innovations in ionization sources such as the concentric hybrid nanoESI-APCI source, couple with chemical derivatization strategies have substantially expanded metabolome coverage, offering a more comprehensive view of the cellular metabolism. Table 1 summarizes the typical MS-based methods for SCM analysis.

**Table 1:** Typical MS-based methods for SCM analysis

MS platform	MS	Cell isolation method/resolution for imaging	Throughput (cell(s) per min)	Coverage (the number of metabolites)	Main metabolites	Ref.
Direct injection-MS	ESI + TOF-MS	Dean flow	∼1	11	Phospholipids	14
	nanoESI + Orbitrap MS	Dean flow	∼40	84	Amino acids, lipids	15
	nanoESI + IMS-TOF MS	Inertial focusing and dean flow	6–8	44	Lipids	57
	PEF-ESI + QE-HF	Inertial focusing	80	∼120	Polar metabolites	16
	Nano-ESI + QE-HF	Inertial focusing	20	213	Glutamine	17
	ESI + QE-HF	Sheath liquid focusing	38	291	Amino acids, lipids, nucleotides	18
	ESI + QE-Orbitrap MS	Sheath liquid focusing	45.3	61	PC	19
	ESI + QE-Orbitrap MS	Sheath liquid focusing	50	69	Lipids	22
	DBDI + MS	Sheath liquid focusing	38	179	Organic acids, lipids	31
	LTP-ESI + QE-HF	Sheath liquid focusing	52	37(50)	Pigments, lipids	37
	nanoESI + QTrap MS	Microfluidic focusing	4	—	PC, amino acids	24
	nanoESI + Orbitrap MS	Dean flow	2.4	∼600	BHB, PC, amino acids	26
	ESI + Orbitrap MS	Sheath liquid focusing	∼50	162	PC, GSH, amino acids	30
	CyESI-MS	Cellular electrical properties	—	10 (differential)	Lipids	42
	ESI + LC-MS/MS	Microfluidic probe isolation	1 cell/3 min (manual)	6 (quantified)	Lipids	43
	ESI + MS	Droplet encapsulation	10 droplets per min	≥10	PC	44
	Pico-ESI + Sincell-100	Size-dependent trap capture	1 cell/90 s	170	Polyamines, amino acids, nucleotides	45
	ILCEI + MS	Equal-diameter ordered capillaries	51	368	PC, nucleotides	27
	ILCEI + IM-MS	Equal-diameter ordered capillaries	25–40	∼800	PC, amino acids	29
	ILCEI + LTQ Orbitrap MS	Equal-diameter ordered capillaries	32	168	Amino acids, nucleotides, PC	74
DESI	DESI + Q-TOF MS	40 µm	—	>100	Lipids	75
	Nano-DESI + MS	∼10 µm	—	284	Lipids	76
	DESI + MRT	35 µm	—	160	Lipids	77
SIMS	HD-MIBI + nano SIMS	30 nm lateral; 5 nm axial	1 cell > 20 h	—	—	63
	GCIB-SIMS	1 µm lateral; 300–400 nm per layer	—	—	Nucleotides	64
	Dual-SIMS	1–3 µm	—	246	Lipids	75
MALDI	MALDI (1,5-DNA) + TOF MS	20 µm	>1000 cells per h	—	Amino acids, PC	61
	MALDI (DHB) + FTICR MS	20 µm	—	>30	Lipids	78

Despite these advances, several interrelated challenges persist. First, the lack of chromatographic separation in typical single-cell MS workflows limits the acquisition of information-rich MS^2^ spectra for structural confirmation, thereby reducing annotation accuracy. Second, absolute quantification remains a major obstacle, as the uniform introduction of internal standards into individual cells is technically challenging, and incomplete cell lysis further undermines accurate abundance estimation. It should be emphasized that the reliable identification and quantitation are the basic requirement of an analytical method, and SCM analysis is no exception. Third, most SCM analyses are conducted under low-salt or non-physiological buffer conditions to ensure MS compatibility, yet the removal of cells from their native microenvironment inevitably perturbs metabolic states, potentially compromising the physiological relevance of the measured metabolite profiles. Additional limitations include limited metabolome coverage, which is often restricted to dozens or hundreds of predominantly high-abundance lipids. Throughput is also insufficient. Current high-speed platforms can be used to analyze only dozens of cells per minute, lagging far behind single-cell transcriptomics. For single-cell spatial metabolomics, balancing spatial resolution and sensitivity remains a fundamental trade-off. Although SIMS achieves subcellular resolution, this often comes at the cost of limited metabolome coverage and reduced ion yield. Furthermore, the complexity of high-dimensional data poses additional challenges for reproducibility and biological interpretation. Due to the significant differences among LC-MS, environmental ionization and MS imaging in terms of sampling and ionization processes, fragment patterns and background features, their metabolic characteristics are usually not directly comparable. This difficulty becomes more pronounced at the single-cell level, where weak signals and system deficiencies complicate cross-platform matching and data integration.

Addressing these challenges will require coordinated efforts across several strategic fronts. Firstly, achieving MS-based SCM under physiologically relevant conditions will rely on the integration of non-invasive sample handling, ambient-compatible ionization technologies, and ultra-sensitive high-speed MS detection. Secondly, next-generation MS instruments such as Waters Xevo™ MRT, SCIEX ZenoTOF 8600 and Thermo Fisher Orbitrap Astral offer attomole-scale sensitivity and acquisition speeds exceeding hundreds of cells per minute, which can significantly improve throughput and detection limits. Very recently, an ultrahigh-throughput platform integrating a flow-focusing ionization source with Xevo™ MRT mass spectrometer was developed to achieve a maximum throughput of 1064.5 cells per min.^73^ With the development of new MS techniques, the identification and quantitation issues of the SCM will be addressed. Thirdly, advances in ion beam technology, high-resolution MSI, and intelligent computational registration will be essential for advancing single-cell spatial metabolomics without compromising data fidelity. Finally, AI-driven computational methods will become increasingly important for processing large and heterogeneous single-cell MS datasets and improving metabolite annotation and in-depth biological interpretation.

In summary, MS-based living SCM stands at the frontier of biological analysis. The next major breakthroughs are likely to arise from the synergistic integration of high-efficiency ionization, intelligent microfluidics, and AI-driven real-time analytics. When combined with stable isotope-labeled substrates and high-resolution MSI, such platforms will enable tracking of nutrient utilization, biosynthetic activity, and metabolic crosstalk between neighboring cells, providing a dynamic complement to static spatial snapshots. In addition, data science will play an increasingly significant role in cross-platform comparison, multi-modal integration, and metabolic phenotype analysis of high-dimensional and sparse single-cell metabolomics datasets. However, many current models are developed under specific analytical conditions and may not perform reliably across platforms or workflows. Future advances will require computational approaches to better integrate analytical constraints, achieve more transferable representations across platforms, and make greater use of reference-based calibration. Through these interdisciplinary advances, the field is expected to evolve from analytical demonstration to the delivery of powerful, biologically insightful tools, ultimately deepening our understanding of life processes and accelerating biomedical translation.

## Author contributions

Xianzhe Shi, Jiajun Peng, Chunxiu Hu and Muyingnan You: writing – original draft, Xin Lu, Xinyu Liu and Guowang Xu: writing – review & editing.

## Conflicts of interest

There are no conflicts to declare.

## Data Availability

No new data are involved in this perspective besides these in the manuscript.

## References

[cit1] Altschuler S. J., Wu L. F. (2010). Cellular heterogeneity: do differences make a difference?. Cell.

[cit2] Lu Y., Xue Q., Eisele M. R., Sulistijo E. S., Brower K., Han L., Amir E.-a. D., Pe’er D., Miller-Jensen K., Fan R. (2015). Highly multiplexed profiling of single-cell effector functions reveals deep functional heterogeneity in response to pathogenic ligands. Proc. Natl. Acad. Sci. U. S. A..

[cit3] Satija R., Shalek A. K. (2014). Heterogeneity in immune responses: from populations to single cells. Trends Immunol..

[cit4] Kumar R., Ghosh M., Kumar S., Prasad M. (2020). Single Cell Metabolomics: A Future Tool to Unmask Cellular Heterogeneity and Virus-Host Interaction in Context of Emerging Viral Diseases. Front. Microbiol..

[cit5] Wang Z., Zhu H., Xiong W. (2023). Advances in mass spectrometry-based multi-scale metabolomic methodologies and their applications in biological and clinical investigations. Sci. Bull..

[cit6] Wang B., Yao K., Hu Z. (2023). Advances in mass spectrometry-based single-cell metabolite analysis. Trends Anal. Chem..

[cit7] Xu T., Feng D., Li H., Hu X., Wang T., Hu C., Shi X., Xu G. (2022). Recent advances and typical applications in mass spectrometry-based technologies for single-cell metabolite analysis. Trends Anal. Chem..

[cit8] Seydel C. (2021). Single-cell metabolomics hits its stride. Nat. Methods.

[cit9] Tsuyama N., Mizuno H., Tokunaga E., Masujima T. (2008). Live single-cell molecular analysis by video-mass spectrometry. Anal. Sci..

[cit10] Fujii T., Matsuda S., Tejedor M. L., Esaki T., Sakane I., Mizuno H., Tsuyama N., Masujima T. (2015). Direct metabolomics for plant cells by live single-cell mass spectrometry. Nat. Protoc..

[cit11] Wei Z. W., Xiong X. C., Guo C. A., Si X. Y., Zhao Y. Y., He M. Y., Yang C. D., Xu W., Tang F., Fang X., Zhang S. C., Zhang X. R. (2015). Pulsed Direct Current Electrospray: Enabling Systematic Analysis of Small Volume Sample by Boosting Sample Economy. Anal. Chem..

[cit12] Huang G. M., Li G. T., Cooks R. G. (2011). Induced Nanoelectrospray Ionization for Matrix-Tolerant and High-Throughput Mass Spectrometry. Angew. Chem., Int. Ed..

[cit13] Wei X., Zhang X., Guo R., Chen M.-L., Yang T., Xu Z.-R., Wang J.-H. (2019). A Spiral-Helix (3D) Tubing Array That Ensures Ultrahigh-Throughput Single-Cell Sampling. Anal. Chem..

[cit14] Huang Q., Mao S., Khan M., Zhou L., Lin J.-M. (2018). Dean flow assisted cell ordering system for lipid profiling in single-cells using mass spectrometry. Chem. Commun..

[cit15] Xu S. T., Liu M. X., Bai Y., Liu H. W. (2021). Multi-Dimensional Organic Mass Cytometry: Simultaneous Analysis of Proteins and Metabolites on Single Cells. Angew. Chem., Int. Ed..

[cit16] Feng D., Li H., Xu T., Zheng F., Hu C., Shi X., Xu G. (2022). High-throughput single cell metabolomics and cellular heterogeneity exploration by inertial microfluidics coupled with pulsed electric field-induced electrospray ionization-high resolution mass spectrometry. Anal. Chim. Acta.

[cit17] Hu X., Liu X., Feng D., Xu T., Li H., Hu C., Wang Z., Liu X., Yin P., Shi X., Shang D., Xu G. (2024). Polarization of Macrophages in Tumor Microenvironment Using High-Throughput Single-Cell Metabolomics. Anal. Chem..

[cit18] Yao H., Zhao H., Zhao X., Pan X., Feng J., Xu F., Zhang S., Zhang X. (2019). Label-free Mass Cytometry for Unveiling Cellular Metabolic Heterogeneity. Anal. Chem..

[cit19] Yang J., Cheng R., Pan X., Pan S., Du M., Yao H., Hu Z., Zhang S., Zhang X. (2024). Single-Cell Unsaturated Lipid Profiling for Studying Chemoresistance Heterogeneity of Triple-Negative Breast Cancer Cells. Anal. Chem..

[cit20] Pan S., Liu C., Yao H., Pan X., Li J., Yang J., Du M., Liu P., Zhang S., Zhang X. (2024). Single-cell metabolite profiling enables information-rich classification of lymphocyte types and subtypes. Chem. Commun..

[cit21] Shen Z., Zhao H., Yao H., Pan X., Yang J., Zhang S., Han G., Zhang X. (2022). Dynamic metabolic change of cancer cells induced by natural killer cells at the single-cell level studied by label-free mass cytometry. Chem. Sci..

[cit22] Shen Z., Yao H., Yang J., Pan X., Zhao H., Han G., Zhang S., Zhang X. (2022). The heterogeneity of oxidized lipids in individual tumor cells reveals NK cell-mediated cytotoxicity by label-free mass cytometry. Analyst.

[cit23] Chen H., Liu J., Zhao Y., Xu H., Liu W., Xing Z., Tang F., Zhang S., Zhang X. (2025). Single-Cell/Particle Sample Introduction Device for Mass Cytometry Based on Gas-Driven Flow Focusing. Anal. Chem..

[cit24] Xu S.-T., Yang C., Yan X.-P. (2021). Nanothorn Filter-Facilitated Online Cell Lysis for Rapid and Deep Intracellular Profiling by Single-Cell Mass Spectrometry. Anal. Chem..

[cit25] Xu S.-T., Yang C., Yan X.-P. (2023). Organic Mass Cytometry Discriminating Cycle Stages of Single Cells with Small Molecular Indicators. Anal. Chem..

[cit26] Qin S., Zhang Y., Shi M., Miao D., Lu J., Wen L., Bai Y. (2024). In-depth organic mass cytometry reveals differential contents of 3-hydroxybutanoic acid at the single-cell level. Nat. Commun..

[cit27] Shao Y., Zhou Y., Liu Y., Zhang W., Zhu G., Zhao Y., Zhang Q., Yao H., Zhao H., Guo G., Zhang S., Zhang X., Wang X. (2022). Intact living-cell electrolaunching ionization mass spectrometry for single-cell metabolomics. Chem. Sci..

[cit28] Zhu G., Zhang W., Zhao Y., Wang G., Yuan H., Guo G., Wang X. (2025). Single-Cell Mass Spectrometry Studies of Secondary Drug Resistance of Tumor Cells. Anal. Chem..

[cit29] Luo M. D., Kou T. Z., Yin Y. D., Zhou S. Y., Zhu X. L., Zeng X. H., Hu J. H., Zhu Z. J. (2026). Deep-coverage single-cell metabolomics enabled by ion mobility-resolved mass cytometry. Nat. Methods.

[cit30] Cheng S. M., Wu D. N., Wang X. X., Tan S. Y., Feng L. L., Yu X. P., Gong X. Y., Dai X. H. (2026). Viability-Informed Single-Cell Mass Spectrometry for More Comprehensive Metabolic Analysis. Anal. Chem..

[cit31] Liu Q., Ge W., Wang T., Lan J., Martínez-Jarquín S., Wolfrum C., Stoffel M., Zenobi R. (2021). High-Throughput Single-Cell Mass Spectrometry Reveals Abnormal Lipid Metabolism in Pancreatic Ductal Adenocarcinoma. Angew. Chem., Int. Ed..

[cit32] Kulyk D. S., Swiner D. J., Sahraeian T., Badu-Tawiah A. K. (2019). Direct Mass Spectrometry Analysis of Complex Mixtures by Nanoelectrospray with Simultaneous Atmospheric Pressure Chemical Ionization and Electrophoretic Separation Capabilities. Anal. Chem..

[cit33] Cheng S.-C., Jhang S.-S., Huang M.-Z., Shiea J. (2015). Simultaneous Detection of Polar and Nonpolar Compounds by Ambient Mass Spectrometry with a Dual Electrospray and Atmospheric Pressure Chemical Ionization Source. Anal. Chem..

[cit34] Liu Q., Lan J., Wu R., Begley A., Ge W., Zenobi R. (2022). Hybrid Ionization Source Combining Nanoelectrospray and Dielectric Barrier Discharge Ionization for the Simultaneous Detection of Polar and Nonpolar Compounds in Single Cells. Anal. Chem..

[cit35] Xu T., Li H., Dou P., Luo Y., Pu S., Mu H., Zhang Z., Feng D., Hu X., Wang T., Tan G., Chen C., Li H., Shi X., Hu C., Xu G. (2024). Concentric Hybrid Nanoelectrospray Ionization-Atmospheric Pressure Chemical Ionization Source for High-Coverage Mass Spectrometry Analysis of Single-Cell Metabolomics. Adv. Sci..

[cit36] Xu Y., Hu X., Yuan Y., Liu W., Wang J., Yang C., Shi X., Qin W., Wen L., Lin M., Jin Y., Wang W., Hu C., Xu G., Wang Q. (2025). Prediction of Lung Cancer Metastasis Risk Based on Single-Cell Metabolic Profiling of Circulating Tumor Cells. Adv. Sci..

[cit37] Yao H., Yang J., Wang Z., Pan X., Pan J., Li H., Zhang S. (2024). High-Throughput Metabolite Analysis of Unicellular Microalgae by Orthogonal Hybrid Ionization Label-Free Mass Cytometry. Anal. Chem..

[cit38] Zhu Y., Wang W., Yang Z. (2020). Combining Mass Spectrometry with Paternò-Büchi Reaction to Determine Double-Bond Positions in Lipids at the Single-Cell Level. Anal. Chem..

[cit39] Li Z., Cheng S., Lin Q., Cao W., Yang J., Zhang M., Shen A., Zhang W., Xia Y., Ma X., Ouyang Z. (2021). Single-cell lipidomics with high structural specificity by mass spectrometry. Nat. Commun..

[cit40] Cheng S., Cao C., Qian Y., Yao H., Gong X., Dai X., Ouyang Z., Ma X. (2024). High-throughput single-cell mass spectrometry enables metabolic network analysis by resolving phospholipid CC isomers. Chem. Sci..

[cit41] Zhuang M., Hou Z., Chen P., Liang G., Huang G. (2020). Introducing charge tag via click reaction in living cells for single cell mass spectrometry. Chem. Sci..

[cit42] Zhu J. W., Pan S. Y., Chai H. C., Zhao P., Feng Y. X., Cheng Z., Zhang S. C., Wang W. H. (2024). Microfluidic Impedance Cytometry Enabled One-Step Sample Preparation for Efficient Single-Cell Mass Spectrometry. Small.

[cit43] Zhang Q., Lin L., Yi X. Z., Xie T. Z., Xing G. W., Li Y. X., Wang X. R., Lin J. M. (2023). Microfluidic Sampling of Undissolved Components from Subcellular Regions of Living Single Cells for Mass Spectrometry Analysis. Anal. Chem..

[cit44] Zhang W. F., Li N., Lin L., Huang Q. S., Uchiyama K., Lin J. M. (2020). Concentrating Single Cells in Picoliter Droplets for Phospholipid Profiling on a Microfluidic System. Small.

[cit45] You J. C., Chen A. Q., Qiu J. X., Ye Y. L., Mei C. C., Fu G. H., Jin B. Y., Wen L. H. (2025). On-chip single-cell mass spectrometry for streamlined metabolic exploration enabled by a reversibly-bonded microfluidic platform. Anal. Chim. Acta.

[cit46] Chen A. Q., Yan M. Y., Feng J. X., Bi L., Chen L., Hu S. D., Hong H. H., Shi L. L., Li G. Q., Jin B. Y., Zhang X. R., Wen L. H. (2022). Single Cell Mass Spectrometry With a Robotic Micromanipulation System for Cell Metabolite Analysis. IEEE Trans. Biomed. Eng..

[cit47] Zhao P., Cheng S. M., Feng Y. X., Liang F., Zhang X. R., Ma X. X., Wang W. H. (2023). Automated and Miniaturized Pico-Liter Metabolite Extraction System for Single-Cell Mass Spectrometry. IEEE Trans. Biomed. Eng..

[cit48] Yang Q. X., Dai Y. B., Huang S. J., Liu B., Sun H. C., Zhou Y., Gong Y. G., Zhu F. (2025). MMEASE: enhanced analytical workflow for single-cell metabolomics. Nucleic Acids Res..

[cit49] Pan X. Y., Pan S. Y., Du M. R., Yang J. L., Yao H., Zhang X. R., Zhang S. C. (2024). SCMeTA: a pipeline for single-cell metabolic analysis data processing. Bioinformatics.

[cit50] Zou Z., Peng Z. K., Bhusal D., Munige S. W., Yang Z. B. (2024). MassLite: An integrated python platform for single cell mass spectrometry metabolomics data pretreatment with graphical user interface and advanced peak alignment method. Anal. Chim. Acta.

[cit51] Liu R. M., Zhang G. W., Sun M., Pan X. L., Yang Z. B. (2019). Integrating a generalized data analysis workflow with the Single-probe mass spectrometry experiment for single cell metabolomics. Anal. Chim. Acta.

[cit52] Sun F. Y., Li H. Y., Sun D. Q., Fu S. L., Gu L., Shao X., Wang Q. Q., Dong X., Duan B., Xing F. Y., Wu J., Xiao M. M., Zhao F. Q., Han J. D. J., Liu Q., Fan X. H., Li C., Wang C. F., Shi T. L. (2025). Single-cell omics: experimental workflow, data analyses and applications. Sci. China Life Sci..

[cit53] Huang L. S., Fang M. L., Cupp-Sutton K. A., Wang Z., Smith K., Wu S. (2021). Spray-Capillary-Based Capillary Electrophoresis Mass Spectrometry for Metabolite Analysis in Single Cells. Anal. Chem..

[cit54] Figueiredo J., Cavaco A. R., Guerra-Guimaraes L., Leclercq C., Renaut J., Cunha J., Eiras-Dias J., Cordeiro C., Matos A. R., Silva M. S., Figueiredo A. (2021). An apoplastic fluid extraction method for the characterization of grapevine leaves proteome and metabolome from a single sample. Physiol. Plant..

[cit55] Zhang H., Ding L., Hu A., Shi X. D., Huang P. H., Lu H. Y., Tillberg P. W., Wang M. C., Li L. J. (2025). TEMI: tissue-expansion mass-spectrometry imaging. Nat. Methods.

[cit56] Sun X. W., Qin A. Z., Wang X. X., Ge X. Y., Liu Z. X., Guo C. X., Yu X. L., Zhang X. L., Lu Y., Yang J. C., He J. M., Zhou Y. P., Liu Y. M., Hu M. K., Liu H., Zhao Z. H., Hu G. J., Li W., Zang X. S., Dai S., Sun S. S., Yong-Villalobos L., Herrera-Estrella L., Tran L. S. P., Ma X. F. (2025). Spatiotemporal transcriptome and metabolome landscapes of cotton fiber during initiation and early development. Nat. Commun..

[cit57] Zhang L., Xu T., Zhang J., Wong S. C. C., Ritchie M., Hou H. W., Wang Y. (2021). Single Cell Metabolite Detection Using Inertial Microfluidics-Assisted Ion Mobility Mass Spectrometry. Anal. Chem..

[cit58] Zhang H., Liu Y., Fields L., Shi X. D., Huang P. H., Lu H. Y., Schneider A. J., Tang X. D., Puglielli L., Welham N. V., Li L. J. (2023). Single-cell lipidomics enabled by dual-polarity ionization and ion mobility-mass spectrometry imaging. Nat. Commun..

[cit59] Qin S., Zhang X., Zhang Y., Miao D., Wei W., Bai Y. (2025). Multi-dimensional bio mass cytometry: simultaneous analysis of cytoplasmic proteins and metabolites on single cells. Chem. Sci..

[cit60] Liu R. M., Li J. N., Lan Y. P., Nguyen T. D., Chen Y. A., Yang Z. B. (2023). Quantifying Cell Heterogeneity and Subpopulations Using Single Cell Metabolomics. Anal. Chem..

[cit61] Zhang Y. Q., Chen P. P., Geng H. Y., Li M., Chen S. P., Ma B. Z., Ma Y., Lai J. J., Cui X. Q., Chong W., Chen H., Wang X., Sun C. L. (2025). Development of a Single-Cell Spatial Metabolomics Method for the Characterization of Cell-Cell Metabolic Interactions. Anal. Chem..

[cit62] Delafiori J., Shahraz M., Eisenbarth A., Hilsenstein V., Drotleff B., Bailoni A., Wadie B., Ekelof M., Mattausch A., Alexandrov T. (2025). HT SpaceM: A high-throughput and reproducible method for small-molecule single-cell metabolomics. Cell.

[cit63] Rovira-Clavé X., Jiang S. Z., Bai Y. H., Zhu B. K., Barlow G., Bhate S., Coskun A. F., Han G. J., Ho C. M. K., Hitzman C., Chen S. Y., Bava F. A., Nolan G. P. (2021). Subcellular localization of biomolecules and drug distribution by high-definition ion beam imaging. Nat. Commun..

[cit64] Pareek V., Tian H., Winograd N., Benkovic S. J. (2020). Metabolomics and mass spectrometry imaging reveal channeled de novo purine synthesis in cells. Science.

[cit65] Lim H., Lee S. Y., Park Y., Jin H., Seo D., Jang Y. H., Moon D. W. (2021). Mass spectrometry imaging of untreated wet cell membranes in solution using single-layer graphene. Nat. Methods.

[cit66] Zhang H., Shi X. D., Lu H. Y., Li L. J. (2025). Delineation of Subcellular Molecular Heterogeneity in Single Cells via Ultralow-Flow-Rate Desorption Electrospray Ionization Mass Spectrometry Imaging. Anal. Chem..

[cit67] Du M., Pan X., Peng Y., Yang J., Pan S., Cheng R., Yang S., Yang Z., Pan J., Liu P., Zhang S., Zhang X. (2025). A Tandem Cytometry Platform for Single-Cell Analysis of Protein and Metabolites. Anal. Chem..

[cit68] Zhao P., Feng Y., Wu J., Zhu J., Yang J., Ma X., Ouyang Z., Zhang X., Zhang W., Wang W. (2023). Efficient Sample Preparation System for Multi-Omics Analysis via Single Cell Mass Spectrometry. Anal. Chem..

[cit69] Lombard-Banek C., Li J., Portero E. P., Onjiko R. M., Singer C. D., Plotnick D. O., Al Shabeeb R. Q., Nemes P. (2021). In Vivo Subcellular Mass Spectrometry Enables Proteo-Metabolomic Single-Cell Systems Biology in a Chordate Embryo Developing to a Normally Behaving Tadpole (X. laevis). Angew. Chem., Int. Ed..

[cit70] He Y., Yuan H., Liang Y., Liu X., Zhang X., Ji Y., Zhao B., Yang K., Zhang J., Zhang S., Zhang Y., Zhang L. (2023). On-capillary alkylation micro-reactor: a facile strategy for proteo-metabolome profiling in the same single cells. Chem. Sci..

[cit71] Wu J., Xu Q.-Q., Jiang Y.-R., Chen J.-B., Ying W.-X., Fan Q.-X., Wang H.-F., Wang Y., Shi S.-W., Pan J.-Z., Fang Q. (2024). One-Shot Single-Cell Proteome and Metabolome Analysis Strategy for the Same Single Cell. Anal. Chem..

[cit72] Mao X., Xia D., Xu M., Gao Y., Tong L., Lu C., Li W., Xie R., Liu Q., Jiang D., Yuan S. (2025). Single-Cell Simultaneous Metabolome and Transcriptome Profiling Revealing Metabolite-Gene Correlation Network. Adv. Sci..

[cit73] Du M. R., Cheng R. S., Chen H. Y., Cao J. Y., Pan S. Y., Yang J. L., Xu K. X., Wang W. J., Ge P. C., Zhang S. C., Zhang X. R. (2026). High-Throughput Single-Cell Lipid Mass Cytometry for Rapid and Robust Screening of Rare Cells. Anal. Chem..

[cit74] Liu Y. X., Zhang W. M., Yuan H. Y., Pei T., Chen T., Gao K., Guo D. H., Yang X. F., Jing N. H., Guo G. S., Wang X. Y. (2026). Exposure single-cell metabolomics mass spectrometry reveals HFPO-DA toxicity mechanisms. Chem. Sci..

[cit75] Tian H., Rajbhandari P., Tarolli J., Decker A. M., Neelakantan T. V., Angerer T., Zandkarimi F., Remotti H., Frache G., Winograd N., Stockwell B. R. (2024). Multimodal mass spectrometry imaging identifies cell-type-specific metabolic and lipidomic variation in the mammalian liver. Dev. Cell.

[cit76] Yin R., Burnum-Johnson K. E., Sun X., Dey S. K., Laskin J. (2019). High spatial resolution imaging of biological tissues using nanospray desorption electrospray ionization mass spectrometry. Nat. Protoc..

[cit77] Vandergrift G. W., Velickovic M., Day L., Gorman B. L., Williams S. M., Shrestha B., Anderton C. R. (2024). Untargeted Spatial Metabolomics and Spatial Proteomics on the Same Tissue Section. Anal. Chem..

[cit78] Levasseur M., Nicol E., Elie N., Houël E., Eparvier V., Touboul D. (2023). Spatialized Metabolomic Annotation Combining MALDI Imaging and Molecular Networks. Anal. Chem..

